# Mechanical Neural Networks with Explicit and Robust Neurons

**DOI:** 10.1002/advs.202310241

**Published:** 2024-06-19

**Authors:** Tie Mei, Yuan Zhou, Chang Qing Chen

**Affiliations:** ^1^ Department of Engineering Mechanics CNMM and AML Tsinghua University Beijing 100084 P. R. China

**Keywords:** artificial neural networks, intelligent mechanical systems, mechanical computing

## Abstract

Mechanical computing provides an information processing method to realize sensing‐analyzing‐actuation integrated mechanical intelligence and, when combined with neural networks, can be more efficient for data‐rich cognitive tasks. The requirement of solving implicit and usually nonlinear equilibrium equations of motion in training mechanical neural networks makes computation challenging and costly. Here, an explicit mechanical neuron is developed of which the response can be directly determined without the need of solving equilibrium equations. A training method is proposed to ensure the robustness of the neuron, i.e., insensitivity to defects and perturbations. The explicitness and robustness of the neurons facilitate the assembly of various network structures. Two exemplified networks, a robust mechanical convolutional neural network and a mechanical recurrent neural network with long short‐term memory capabilities for associative learning, are experimentally demonstrated. The introduction of the explicit and robust mechanical neuron streamlines the design of mechanical neural networks fulfilling robotic matter with a level of intelligence.

## Graphical abstract

An explicit and robust mechanical neuron without the need is proposed to solve the inherent implicit governing equations, facilitating the design of mechanical neural networks. Two examples are provided: a mechanical convolutional neural network that can recognize handwritten digits even if some components are removed, and a mechanical recurrent neural network with long short‐term memory capabilities that mimics Pavlov's dogs.

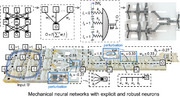

## 1 Introduction

The growing demand for matter with intelligence is driving the development of unconventional information processing methods, including mechanical computing.^[^ ^1^, ^2^ ^]^ Different levels of mechanical computing have been developed, such as mechanical logic gates,^[^ ^3^, ^4^, ^5^ ^]^ mechanical memory units,^[^ ^6^, ^7^, ^8^ ^]^ signal propagation strategies,^[^ ^9^, ^10^ ^]^ mechanical logical circuits,^[^ ^11^, ^12^, ^13^ ^]^ and mechanical computing architectures.^[^ ^14^, ^15^, ^16^ ^]^ Though limited in performance compared to silicon‐based electronic computing, mechanical computing offers unique potentials associated with data security and resistance to electromagnetic interference,^[^ ^17^, ^18^, ^19^ ^]^ and easy‐to‐be integrated with materials.^[^ ^20^ ^]^ In addition, with recent advances in materials and fabrication techniques,^[^ ^3^, ^21^ ^]^ mechanical computing systems can process environmental information in a sensing‐analyzing‐actuation integrated manner, thus promoting the development of intelligent mechanical systems such as soft robotics and metamaterials.^[^ ^20^, ^22^ ^–^ ^25^ ^]^

Artificial neural networks (ANNs)^[^ ^26^, ^27^ ^]^ have achieved notable success in performing complex information processing tasks, such as computer vision^[^ ^28^ ^]^ and machine translation.^[^ ^29^ ^]^ Combining mechanical computing with artificial neural networks is expected to enhance the intelligence of mechanical systems further. Similar initiatives have been explored in other physical systems by identifying a mapping between the physical input‐output relationship and the commonly used mathematical models of ANNs, leading to the development of physical neural networks. For instance, optical matrix multipliers^[^ ^30^ ^]^ and the optical Kerr effect^[^ ^31^ ^]^ can mimic the linear weighted summation and nonlinear activation functions of an artificial neuron and have been employed to develop optical neural networks^[^ ^32^ ^]^; wave‐based systems, based on the dynamics of waves have been shown to mirror the computations in recurrent neural networks^[^ ^33^ ^]^ (RNNs), paving the way for wave‐based RNNs.^[^ ^34^, ^35^ ^]^ In addition, leveraging waves passing through successive physical substrates can facilitate information processing akin to deep neural networks.^[^ ^36^, ^37^ ^]F^

For mechanical neural networks (MNNs),^[^ ^38^, ^39^, ^40^ ^]^ the input, i.e., the displacement or force loads applied to the mechanical nodes, drives the deformation of the whole network and the information processing process. The network evolves to the position of the elastic energy minima representing the output. However, the geometrical‐ and material‐parameter (i.e., the weights in MNNs) dependent input‐output relationship, in general, can hardly be expressed explicitly as that in commonly used artificial neural networks and can only be inferred by solving the implicit nonlinear equilibrium equations of motion. Solving equilibrium equations is thus a prerequisite for the design of MNNs, which is hindered by the involved computational inefficiency.^[^ ^41^ ^]^ The inefficiency is especially true when a large number of mechanical nodes are involved, and renders it hard to design MNNs based on more advanced ANN models like convolutional neural networks^[^ ^42^ ^]^ (CNNs), RNNs, and long‐short term memory^[^ ^43^ ^]^ (LSTM).

Function‐complete explicit mechanical neurons that respond to input in accordance with an explicit expression composed of linear weighted summation and nonlinear activation operations are thus highly desirable. However, most available basic mechanical computing modules are non‐neuromorphic logic gates (e.g., AND, OR, NOT gates).^[^ ^3^, ^4^, ^9^, ^44^, ^45^ ^]^ Constructing a mechanical neuron with these logic gates often requires the assembling of multiple different gates,^[^ ^46^ ^]^ which increases the number of components of a mechanical neuron and impedes the realization of more advanced ANN models based MNNs.

A recently proposed in‐memory mechanical computing architecture^[^ ^16^ ^]^ allows mechanical data to be processed in a neuromorphic manner. Leveraging this architecture, an explicit mechanical neuron equivalent to the McCulloch–Pitts model^[^ ^47^ ^]^ is explored in this study, enabling weighted summation of inputs followed by non‐linear activation without assembling logic gates. With the explicit neuron, the output of an MNN can be derived explicitly from the input, simplifying the design process of MNNs without the need of solving equilibrium equations.

In addition, mechanical systems are vulnerable to defects induced either during production or in‐service damage. For the mechanical neuron related to an analog computation process of weighted summation, this problem will become even more worse, compared with the mechanical logic gates^[^ ^3^, ^4^, ^9^, ^44^, ^45^ ^]^ that are intrinsically digital. Thus, it is important to develop a training method for constructing robust MNNs to resist weight perturbations. Then, applications of the developed robust and explicit mechanical neurons in assembling MNNs based on two types of more advanced ANN models are given. The first is a mechanical convolutional neural network (MCNN), wherein the sharing weights reduce the number of mechanical connections on a node and can further enhance the robustness of the network. The MNN can successfully perform a handwritten digit recognition task even if some springs are removed to mimic the weight perturbations. The second is a mechanical recurrent neural network (MRNN) integrated with LSTM capabilities through sub‐networks like mechanical loops and the input, forget, and output gates. This MRNN supports the function of a Pavlov's dog that learns in real‐time.

## 2 Results

### 2.1 Construction of Explicit Mechanical Neurons

**Figure 1:**
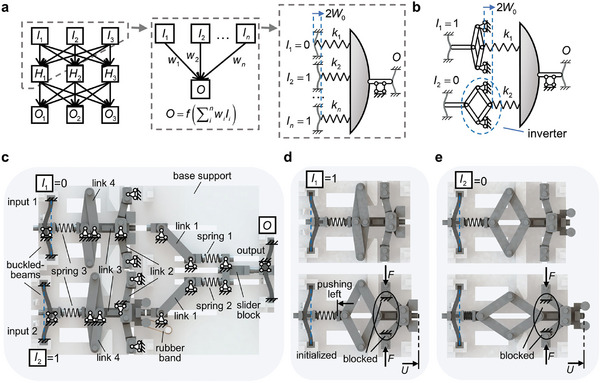
Buckled beam‐based explicit mechanical neuron. a) Schematic of a mechanical neural network consisting of buckled beam‐based neurons. b) Buckled beam‐based mechanical neuron containing an inverter. c) Structural design of the mechanical neuron with two inputs. An inverter is added to the input 2. d) The input buckled beam *I*_1_ in state 1 gives a displacement load *U* toward the output beam *O* driven by the external force *F* during computation. e) The input buckled beam *I*_2_ in state 0 gives a displacement *U* toward the output beam *O* driven by the external force *F*.

The basic computational module of artificial neural networks is artificial neurons that compute the linear weighted (*w_i_*) summation of the inputs (*I_i_*) and produce an output (*O*) with a subsequent non‐linear activation function (*f*(·)) (**Figure** **1**a, left). Here, a mechanical structure (i.e., mechanical neuron) consisting of bi‐stable buckled beams and springs is constructed to mimic an artificial neuron, i.e., an MNN (Figure 1a, right).

There are two stable states for the buckled beam. It can thus be used as a non‐volatile mechanical memory unit^[^ ^6^ ^]^ to store the binary bits 0 or 1 as the beam stays at different stable states, i.e., arching to the left or right. The stored binary bits in the left buckled beam are the inputs while those in the right buckled beams are the output. A series of parallel springs are used to connect to the input and output buckled beams. When the *i*‐th input beam is in state 1 (0), i.e.,*I_i_* = 1(0), the corresponding spring of stiffness *k_i_* will be pushed for a distance of *U* and thus the output beam is pushed. If the sum of the stiffnesses of the pushed springs, i.e., $\sum_{i=1}^{n}I_{i}k_{i}$ where *n* is the number of springs, is greater than the critical stiffness k*, the beam will be pushed to state 1. Therefore, Iiki/Iikik∗k∗ can represent the compression strength applied to the output beam from the *i*‐th input. Then, considering the weights as wi=ki/kik∗k∗, the output can be written as a step function (ε(*x*) = 0, if *x* < 0; ε(*x*) = 1, if *x* ≥ 0) of the weighted summation of the inputs as: $O=\varepsilon (\sum_{i=1}^{n}w_{i}I_{i}-1)$, which is equivalent to the classic Mcculloch–Pitts neuron model. The detailed mechanism of the mechanical neuron will be discussed in the Experimental Section.

Note that the weights of the mechanical neuron can only be set as a series of positive values derived from the stiffness of given springs, i.e., *k_i_*/k* (*k_i_* > 0), which limits the capability of MNN. To expand the weight space, an inverter can be introduced into the mechanical neuron (Figure 1b). When the input is 1, e.g., *I*_1_ = 1, the corresponding spring is not compressed. When the input is 0, e.g., *I*_2_ = 0, the links in the inverter are opened and the spring is compressed. The compression strength applied to the output beam can be represented as (1−Ii)ki/(1−Ii)kik∗k∗. The minus sign before *I_i_* introduces negative weights in the mechanical neuron. The input‐output relationship of the neuron in Figure 1b is $O=\varepsilon (\sum_{i=1}^{2}w_{i}\left(I_{i}-1\right)-1)$, with wi=−ki/kik∗k∗. In doing so, the weight space is expanded to include both positive and negative weights to support functional‐complete responses. In addition, by adding an input *I_b_* = 1 and the corresponding spring *k_b_*, a bias can be introduced to the mechanical neuron. The bias space can be extended to include both positive and negative values by changing the geometry of the buckled beam (see the Experimental Section). Now, the forward process of the buckled beam‐based neuron can be written explicitly as:

(1) $$O=\varepsilon \left(\sum_{i=1}^{n}w_{i}I_{i}-\sum_{w_{i}<0}w_{i}+b-1\right)$$

which is the base for robustness analysis and constructing MNNs of more advanced ANN models.

For the experimental realization of the buckled beam‐based mechanical neuron, the base support and links are added to the structural design (Figure 1c). More details of fabrication can be found in the Experimental Section. There are two input and one output buckled beams, and the corresponding links, with their boundary conditions shown in Figure 1c. The link 2 related to input 1 arches to the left when *I*_1_ = 0 (Figure 1c, top). If the input 1 is changed to 1, the spring 3 will be pushed thus the related links 2 to 4 move right, and the link 2 arches to the right (Figure 1d, top). During computing, link 2 is compressed vertically by a pair of external forces *F* provided by a magnet. Considering the arching direction of link 2, it generates a displacement load *U* on spring 1 toward the output beam only if *I*_1_ = 1 (Figure 1d, below). Note that the rhombic‐shaped link 3 also opens, and thus pushes the spring 3 and initializes the input beam to state 0.

To expand the weight space, there is a change in geometric design for the links related to input 2, and it arches to the left when *I*_2_ = 1 (Figure 1c, below). After changing the input 2 to state 0, link 2 will be pulled by the rubber band and arch to the right (Figure 1e, top). Consequently, an inverter is embedded to input 2 and the corresponding link 2 will generate a displacement load *U* on spring 2 toward the output beam only if *I*_2_ = 0 (Figure 1e, below). Then, the compression strength applied to the output beam can be represented as (1−I2)k2/(1−I2)k2k∗k∗, where the minus sign before *I*_2_ introduces negative weights in the mechanical neuron. The input‐output relationship of the neuron in Figure 1c is: *O* = ε(*w*_1_ *I*_1_ + *w*_2_ *I*_2_ − *w*_2_ − 1), w2=−k2/k2k∗k∗. By introducing more inputs and the bias the output of the buckled beam‐based mechanical neuron can be written as Equation (1).

In addition to the buckled beam‐based mechanical neuron, other explicit mechanical neurons are also possible. Two additional mechanical neurons are discussed in the Supplementary Information. One consists of a slider on a rough horizontal plane and a series of parallel springs. Another is constructed by intersecting water pipes. These examples show that MNNs with explicit neurons can be realized in a variety of mechanical platforms with different nonlinear activation functions (e.g., the rectified linear unit abbreviated as ReLU). Higher information density and environmental adaptability can be achieved by choosing suitable mechanical neurons though here we focus on the buckled beam‐based MNN. The idea can also be applied to other types of neurons and thus provides a paradigm for constructing MNNs without solving implicit equations.

### 2.2 Training for Robust Mechanical Neural Networks

We now introduce a training method to obtain not only explicit but also robust mechanical neurons and thus the corresponding robust MNN to benefit its practical applications. To train an MNN, a loss function differentiating the target output *O_t_* and actual output *O* can be defined as L1=(O−Ot)2/(O−Ot)222. The target weights and biases for the desired input‐output relationship are searched along the gradient descent direction of the loss function. For the aforementioned buckled beam based neuron, the training process is explicit, no implicit nonlinear equations are solved (see the Experimental Section). Thus, the explicit MNN can be trained more efficiently. In addition, the training method for the commonly used artificial neural networks of the machine learning community can be directly applied to the MNN in this paper, which is assured by the mathematical equivalence between these two types of networks.

It should be pointed out that owing to manufacturing scatter, or defects either inherent to fabrication or induced during in‐life service, an inevitable discrepancy between the actual weights in a mechanical system and the ideal ones exists (for example, see the scatter in Figure S1e, Supporting Information). The discrepancy can lead the MNN to lose its target input‐output relationship and not function as expected. Thus, it is necessary to account for the robustness of the MNN during training, i.e., the resistance to weight perturbations.

A necessary condition for the MNN to lose its target input‐output relationship is that, under weight perturbations, the output of at least one neuron in the network deviates from its expectation. Thus, it is important to improve the robustness of mechanical neurons to improve the robustness of a network. For a mechanical neuron, considering the step function mutates at 0, the farther the weighted summation of the *k*‐th neuron $a^{\left(k\right)}=\sum_{i=1}^{n^{\left(k\right)}}w_{i}^{\left(k\right)}I_{i}^{\left(k\right)}-\sum_{w_{i}<0}w_{i}^{\left(k\right)}+b^{\left(k\right)}-1$ is from 0, the more difficult it is for the output to change under perturbation. To show this viewpoint, assume that the weight perturbation for all the weights and biases are the same (denoted by Δ*w*). The weights and bias become $w_{i}^{\left(k\right)}+\Delta w$ and $b_{i}^{\left(k\right)}+\Delta w$, and the change in the weighted summation is $|\Delta a^{\left(k\right)}\left|=\right|\sum_{i=1}^{n^{\left(k\right)}}\Delta wI_{i}^{\left(k\right)}-\sum_{w_{i}^{\left(k\right)}<0}\Delta w+\Delta w|$. When the output of the mechanical neuron does not match the target under the perturbation, one has |Δ*a* ^(*k*)^| > |*a* ^(*k*)^|. Consequently, the weight perturbations for the neuron to lose its target output can be evaluated as:

(2) $$\left|\Delta w\right|>\frac{\left|a^{\left(k\right)}\right|}{\left|\sum_{i=1}^{n^{\left(k\right)}}I_{i}^{\left(k\right)}-\sum_{w_{i}^{\left(k\right)}<0}1+1\right|}\geq \frac{\left|a^{\left(k\right)}\right|}{n^{\left(k\right)}+1}$$

where $\sum_{i=1}^{n^{\left(k\right)}}I_{i}^{\left(k\right)}-\sum_{w_{i}^{\left(k\right)}<0}1=n^{\left(k\right)}$ when all the weights are larger than 0 ($w_{i}^{\left(k\right)}>0$) and the inputs are all 1 ($I_{i}^{\left(k\right)}=1$). It can be found that the smaller *n* ^(*k*)^ or the larger |*a* ^(*k*)^|, the higher the weight perturbation |Δ*w*| to make the neuron mismatch the target, i.e., the higher the robustness. In terms of training, the number of connections on a node is pre‐selected and the robustness is enhanced by increasing the minimal absolute value of the weighted summation, i.e., min (|*a* ^(*k*)^|), for all the neurons of the MNN.

Accordingly, apart from the loss function *L*_1_, an additional loss function *L*_2_ can be defined, i.e.,

(3) $$L_{2}=\frac{1}{N}\sum_{k=1}^{N}{\tilde{L}}_{2}^{\left(k\right)},{\tilde{L}}_{2}^{\left(k\right)}=\left\{\begin{array}{c} {\left(a^{\left(k\right)}-r\right)}^{2}/2,0<a^{\left(k\right)}\leq r\\ {\left(a^{\left(k\right)}+r\right)}^{2}/2,-r<a^{\left(k\right)}\leq 0\\ 0,\left|a^{\left(k\right)}\right|\geq r \end{array}\right.$$

where *N* is the number of neurons, *r* is defined as the robustness parameter. By minimizing *L*_2_, *r* becomes the lower bound of |*a* ^(*k*)^| for all the mechanical neurons. In doing this, the robustness can be adjusted by setting different values *r*. The larger the *r*, the more robust the MNN.

Furthermore, it is hard to fabricate mechanical components that correspond to any value of weight and bias. For this reason, we use the standard, commercially available springs. Thus, the candidate weight and bias can only be selected from a series of specific discrete values. Neural networks with discrete weights have already been studied.^[^ ^48^, ^49^ ^]^ These networks can reduce memory usage and are especially promising for developing deep models on resource‐limited devices. We generalize the training method in^[^ ^49^ ^]^ to train the buckled beam‐based MNN with the weight‐bias set consisting of discrete values. The network is first trained with continuous weights. Then, the weights are discretized and updated while letting the network be aware of the additional error introduced by discretization. More details of the training process to enhance the robustness of the MNN by selecting the weights and bias from specific discrete values are given in the Experimental Section.

**Figure 2:**
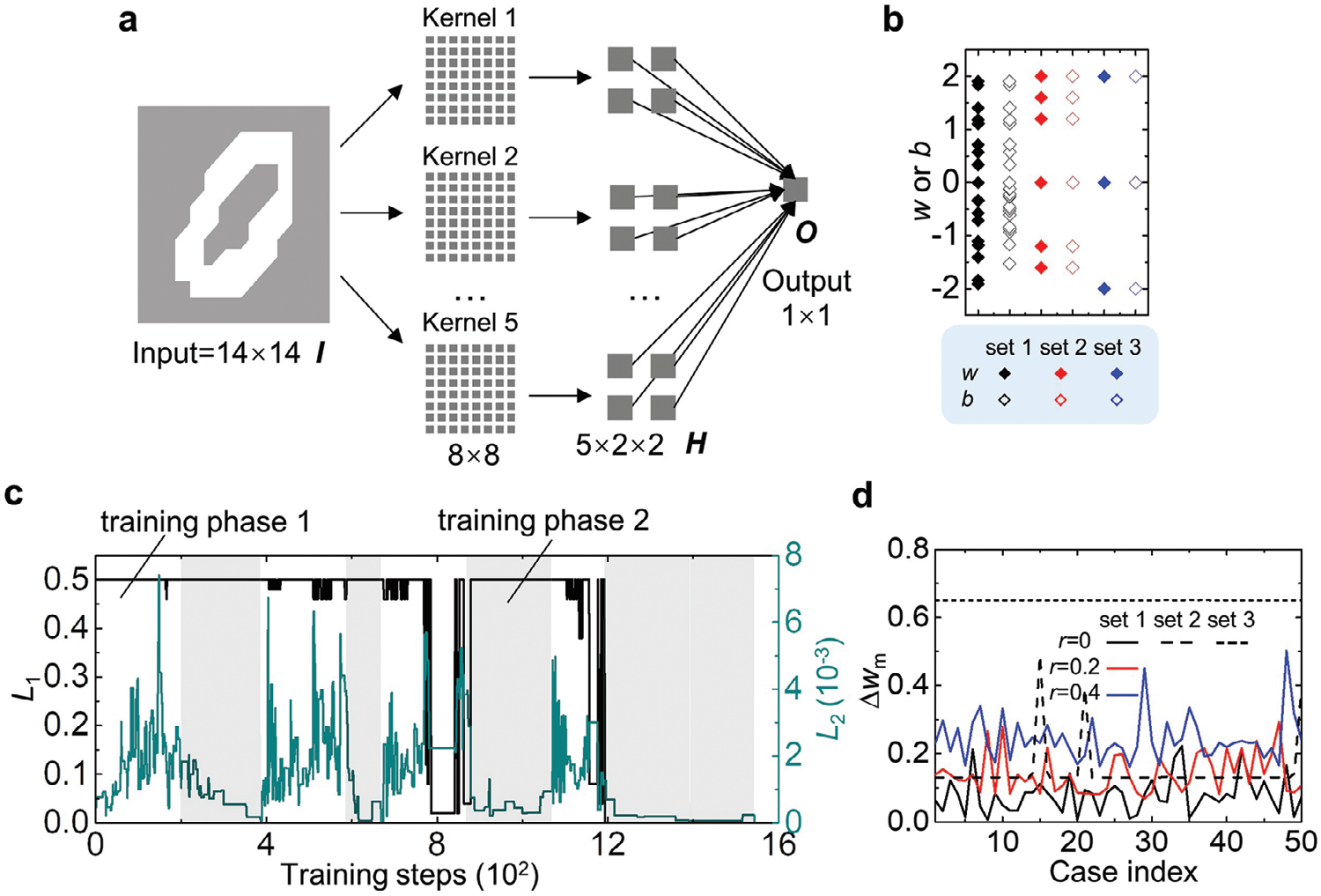
Training of the robust mechanical neural network. a) A neural network trained to distinguish handwritten numbers. b) The set for discrete weights and biases. c) The training process is informed of the constraint of discrete weights and biases of set 1 and high robustness of *r* = 0.4. d) The minimum weight perturbation that makes the network fail to distinguish all handwritten numbers for different weight‐bias sets and robustness parameters *r*.

As an exemplified application, we train an MNN in MATLAB for the recognition of handwritten numbers with 50 ‘0’s and 50 ‘1’s randomly chosen from the MNIST database.^[^ ^50^ ^]^ The training set consists of 100 handwritten digits (including 50 handwritten zeros and 50 handwritten ones. The learning rate is 0.1. The structure of the network is shown in **Figure** **2**a, including an input layer of 14 × 14 nodes, a hidden layer of 5 × 2 × 2 nodes, and an output layer of one node. The input and hidden layers are connected by five 8 × 8 convolution kernels, and the output node is connected to all the hidden nodes. The trained network is supposed to output 0 (1) when the input is a handwritten ‘0′ (‘1′). During training, the weight‐bias set of 1 is used (shown in Figure 2b), which is obtained with the springs shown in Figure 2g. When searching the target weights in the set of discrete values, a greedy approach is used (Figure 2c, *r* = 0.4): two computing phases are conducted alternately and the loss functions *L*_1_ and *L*_2_ are minimized, respectively, until reaching a maximum number of iteration steps of 200. Finally, these two loss functions are both minimized to 0, i.e., the network can recognize all the handwritten numbers and the robustness is also enhanced.

For a given *r*, we train the MNN 50 times with randomly selected initial weights, biases, and handwritten digits. To test the robustness of the trained MNN, a random perturbation of Δ*w* is added to all weights and biases, and the minimum Δ*w* (denoted as Δ*w_m_*) to make the network fail to recognize all the handwritten numbers is recorded to represent the robustness. The greater the robustness, the larger Δ*w_m_*. All the records Δ*w_m_* are shown in Figure 2d. It can be found that Δ*w_m_* can be very small (≈0.001) when *r* = 0 (i.e., the black solid line, trained without enhancing robustness). This indicates that the network may lose its expected function even with a very small defect, making it difficult for the network to be used in practice. Comparing the black, red, and blue solid lines, the training method does work and provides a strategy to adjust the robustness, i.e., the larger *r* the higher the robustness. The weight perturbation produced as the maximum standard deviation of the springs in Figure S1e (Supporting Information) is ≈0.08. When setting *r* = 0.4 (the blue solid line), the minimum Δ*w_m_* is ≈0.16, far greater than 0.08, which indicates the network is robust enough against possible defects.

As an alternative training method to enhance robustness, the network can be trained with a weight‐bias set composed of deliberately selected discrete values. If the weights and biases are chosen from this set, the lower bound of |*a* ^(*k*)^| for all neurons will be high, and so is the robustness. For example, two deliberately designed weight‐bias sets (sets 2 and 3) are given in Figure 2b. Set 2 is [−1.6, −1.2, 0, 1.2, 1.6, 2] and set 3 is [−2, 0, 2]. It can be verified that the absolute value of the weighted summation |*a* ^(*k*)^| for the two sets is always larger than 0.2 and 1, respectively. Accordingly, the robustness of the corresponding MNN is equal to those trained with *r* = 0.2 and *r* = 1. As shown by the black dash and dot lines in Figure 2d, the robustness of the MNN trained with the weight‐bias sets 2 and 3 can be improved even if *r* = 0. As a rule of thumb for the choice of the weight‐bias set with enhanced robustness, the set can be $\left[-1-\tilde{r},0,1+\tilde{r}\right]$, ($\tilde{r}>0$). If the weights and biases belong to this set, $|a^{\left(k\right)}+1\left|=\right|\sum_{i=1}^{n^{\left(k\right)}}w_{i}^{\left(k\right)}I_{i}^{\left(k\right)}-\sum_{w_{i}<0}w_{i}^{\left(k\right)}+b^{\left(k\right)}|$ belongs to $\left[0,1+\tilde{r},2\left(1+\tilde{r}\right),3\left(1+\tilde{r}\right),\text{…}\right]$ while $I_{i}^{\left(k\right)}$ = 0 or 1. Thus, $|a^{\left(k\right)}|\geq \min\left(\tilde{r},1\right)$. By setting a larger $\tilde{r}$, the lower bound of |*a* ^(*k*)^| will be higher when $\tilde{r}\leq 1$, and consequently, the trained MNN becomes more robust.

### 2.3 Mechanical Convolutional Neural Networks

In the following, we show experimentally the method for training a robust network with a weight‐bias set of discrete values. We also show the design of network structures composed of explicit and robust mechanical neurons for two examples, i.e., an MCNN and an MRNN.

Convolutional neural networks (CNN),^[^ ^42^ ^]^ by the sharing of weights and sub‐sampling in the network, can reduce the memory requirements and are equipped with the invariance to small geometric transformations or distortions, which is of great benefit in image or speech recognition. By assembling the explicit and robust mechanical neurons while sharing mechanical connections, a MCNN can be obtained. Note that the number of mechanical connections on a node is reduced by sharing weights (i.e., *n* ^(*k*)^ becomes smaller) and according to Equation (2), the network's robustness is enhanced. Moreover, the robustness of the MCNN can be further improved by adopting the robust training method introduced before.

**Figure 3:**
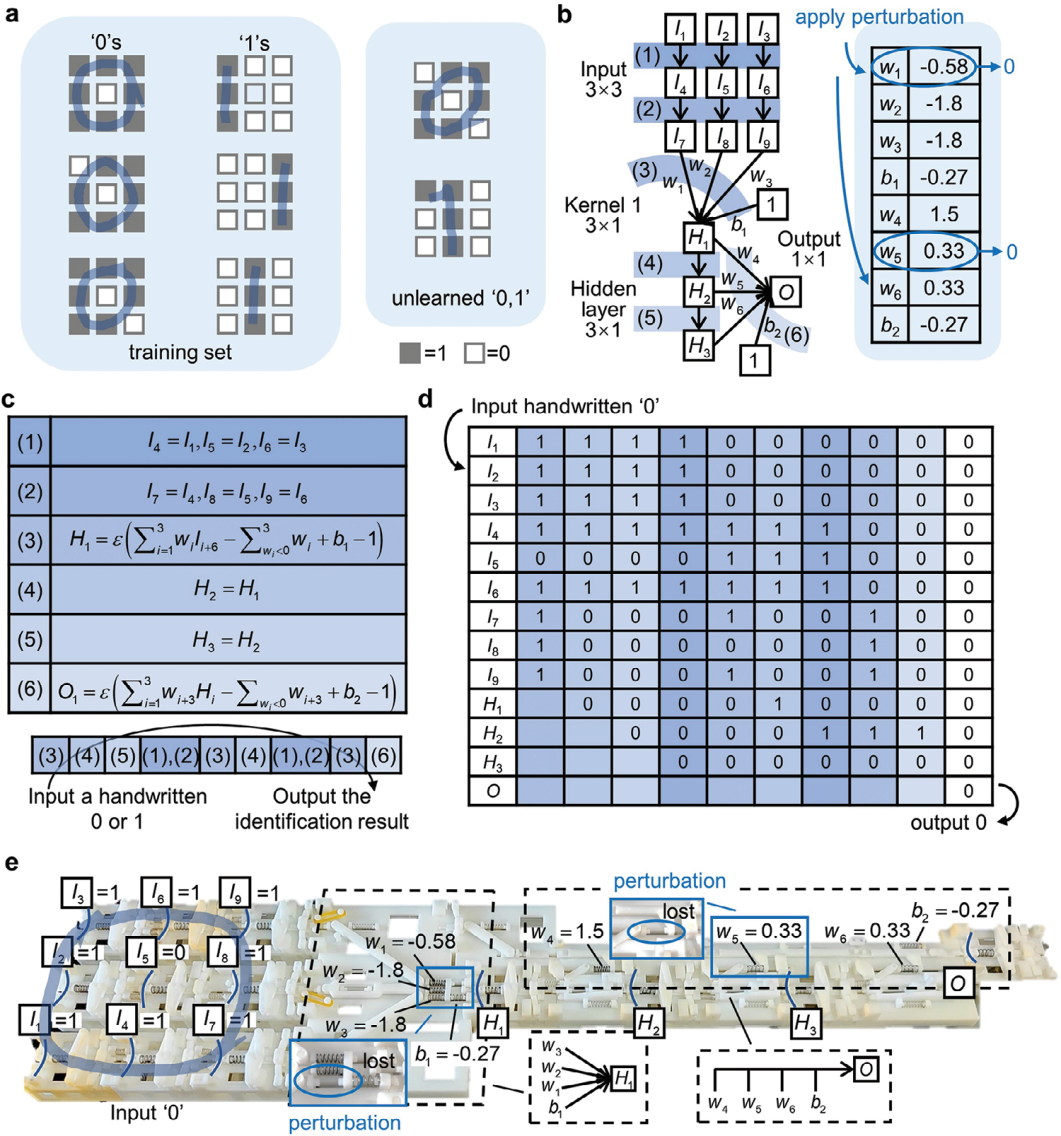
A robust mechanical convolutional neural network (MCNN) for the recognition of handwritten digits. a) The handwritten digits ‘0’ and ‘1’ of 3 × 3 bits. b) Schematic illustration of a MCNN with 3 × 3 nodes in the input layer, three nodes in the hidden layer, and one node in the output layer. When the weights are set as the right table, the network is robust against perturbation in weight and bias. c) The math operations in a working period of the network. d) Computing process as the state evolution of all the nodes when the input is a handwritten ‘0’. e) The experimental system of MCNN.

An MCNN for robustly recognizing handwritten digits is shown in **Figure** **3**. The digits are handwritten ‘0’s and ‘1’s composed of 3 × 3 mechanical bits (Figure 3a). Two additional unlearned digits are prepared to test the capability of generalization of the network. A schematic diagram of the MCNN is given in Figure 3b, where the square blocks are mechanical bits made of a buckled beam, each representing a binary bit (e.g., *I_i_* = 0 or 1). The black arrow represents a shift register operation, i.e., the binary state of the mechanical bits before the arrow is transferred to the bits after the arrow. The intersecting arrows with a weight parameter *w_i_* represent the mechanical neuron of Equation (1). The network is composed of an input layer of 3 × 3 nodes, a hidden layer of 3 × 1 nodes, and one output node. The hidden layer is connected to the input by a convolutional kernel of 3 × 1 weights while the output node is connected to all the hidden nodes. The network is trained with the method given in the previous section with the weight‐bias set of 1 and *r* = 0.5. The network is supposed to output 0 (1) when the input handwritten digit is 0 (1). The trained weights are listed (Figure 3b, right). To test the robustness of the MNN, the minimum weights in the hidden and output layers *w*_1_, *w*_5_ will be artificially set to 0 (by removing the corresponding springs).

The math operations in Figure 3b are divided into six groups. Their mathematical expressions are listed in Figure 3c. The whole process to recognize a handwritten digit is also given. As an example, the binary state evolution of all the mechanical bits during recognizing a handwritten ‘0’is given in Figure 3d. The network outputs a 0. During the process, the mechanical bits in different rows of the input layer are successively processed and the results are stored to the corresponding mechanical bits in the hidden layers with the help of shift register operation, i.e., the results of $\varepsilon (\sum_{i=1}^{3}w_{i}I_{i+3j}-\sum_{w_{i}<0}w_{i}+b_{1}-1)$ is stored to *H*_*j* + 1_, *j* = 0, 1, 2. Then, the mechanical bits in the hidden layer are processed and an output is obtained. The experimental system of the MCNN is shown in Figure 3e, wherein the input is a handwritten ‘0’, the relationship of several typical parts given by the schematic diagram in Figure 3b is marked, the values of the weight and bias are listed, and the artificially removed springs during the robustness test are also marked. Video S1 (Supporting Information) shows the corresponding computing process of the MCNN. It is found that the trained network successfully recognizes all the handwritten digits including the unlearned ones. Moreover, after removing the springs marked in Figure 3e, the MNN can still recognize the handwritten digits, a signal of the robustness of the trained MCNN (Video S2, Supporting Information).

Apart from the advantage in constructing robust networks, the handwritten digits ‘1’s in Figure 3a indicate the translational invariance of the MCNN. With the convolution operation, the number of mechanical connections is greatly reduced. For example, there are only three connections between the input and hidden layers in Figure 3e, while there would be 27 connections if these nodes are fully connected. The reduction in the number of connections not only reduces the physical interference of the connections, but also eases fabrication of the mechanical components.

### 2.4 Mechanical Recurrent Neural Networks with Long Short‐Term Memory

**Figure 4:**
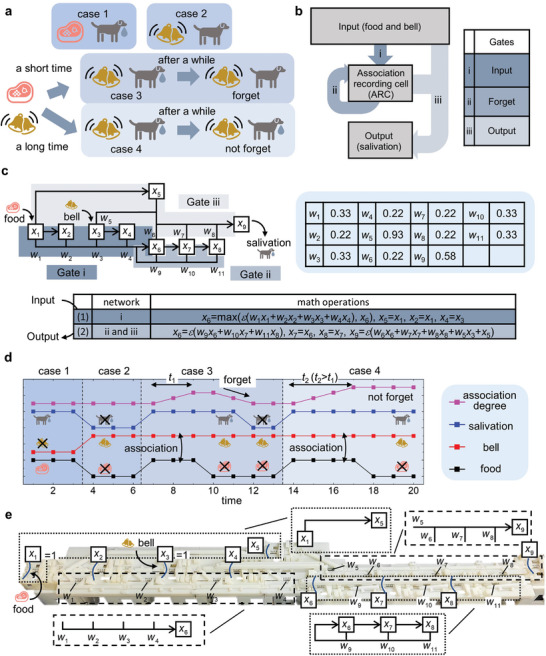
A mechanical recurrent neural network with long short‐term memory. a) Four typical cases for Pavlov's dog. b) A network structure to mimic Pavlov's dog in a). c) Schematic illustration of MRNN, together with the weights and the math operations during a working period. d) The state changing of the nodes corresponding to the ‘food’, ‘bell’, ‘salivation’, and the association degree for cases 1–4. e) The experimental system of a mechanical Pavlov's dog with long short‐term memory.

To further demonstrate the potential of the explicit and robust mechanical neuron in designing MNNs, another type of MNN with more advanced network structure, i.e., an MRNN, is constructed. Recurrent neural networks (RNNs)^[^ ^33^ ^]^ are embedded with a looping mechanism that allows information to persist over time. They are designed to process and predict sequential data and are widely used in language modeling and speech recognition. To improve the performance of RNNs in the processing of long sequential data, the LSTM architecture^[^ ^43^ ^]^ is proposed by introducing sub‐structures, including the input, forget, and output gates, as well as the memory cell. By suitable combination of the explicit neuron, sub‐structures for MNNs, e.g., the loop, the gates, and so on, can also be designed. In the following, an MRNN with long short‐term memory can be constructed to mimic Pavloʼs dog that learns in real‐time (**Figure** **4**).

There are four typical responses of a Pavlov's dog, summarized as cases–4 (Figure 4a). In case 1, the ‘dog’ salivates when it sees the ‘food’. In case 2, the ‘dog’ does not salivate when the ‘bell’ rings. In case 3 (short‐term memory), if the ‘bell’ rings for a while at the same time as the ‘dog’ sees the ‘food’, the ‘dog’ will salivate even if it only hears the ‘bell’. However, after a while, it will forget the association between the ‘food’ and the ‘bell’ and will not salivate when the ‘bell’ rings. Furthermore, if the ‘bell’ and ‘food’ are present simultaneously long enough, the ‘dog’ will not forget their association and will salivate when it hears the ‘bell’, no matter how long it has been (Case 4, long‐term memory).

To mimic all the typical response cases, a MRNN is designed, Figure 4b. The network can be divided into three parts, i.e., the input for sensing and recording the ‘food’ and ‘bell’ signal, the association recording cell (ARC), and the output to provide the ‘salivation’ signal. The input is connected to the ARC with the input gate. This gate is embedded with a user‐defined criterion to determine the degree of association. If the ‘food’ and ‘bell‘ signals meet the criterion, the degree of association stored in the ARC will increase. The ARC is equipped with a recurrent loop serving as the forget gate. With this gate, the degree of association will remain at the current level to form long‐term memory if certain conditions are met. Otherwise, it will decrease as time goes on (short‐term memory). In addition, the output and ARC are connected to the output through the output gate. The gate will output the ‘salivation’ signal when the ‘food’ signal is input or when the ‘bell’ signal is input and the degree of association is high enough.

A schematic diagram of the MRNN is given in Figure 4c. The mechanical bits *x*_1_ and *x*_3_ are used for sensing the ‘food’ and ‘bell’ signals, while *x*_2_ and *x*_4_ are used to store these signals. The mechanical bits *x*_6_, *x*_7_, and *x*_8_ represent the degree of association as *x*_6_ + *x*_7_ + *x*_8_. And the state of *x*_9_ represents the ‘salivation’ signal. The weights are listed on the right of Figure 4c. The network learns in real‐time with one work period after another. During a working period, the network receives the input and outputs the ‘salivation’ signal after two steps (listed in Figure 4c, below). In the first step, the ‘food’ signal is transmitted and stored to the mechanical bits of *x*_2_ and *x*_5_ while the ‘bell’ signal is transmitted and stored to *x*_4_. In addition, *x*_6_ will be calculated as 1 if *x*_1_ = *x*_2_ = *x*_3_ = *x*_4_ = 1, i.e., the ‘food’ signal is present with the ‘bell’ signal in both the current and previous working periods. In doing so, the network establishes the association between these two signals, and the degree of association stored in ARC increases. In the second step, *x*_9_ will be calculated as 1 (outputting the salivation signal) when *x*_5_ = 1 (i.e., there is a ‘food’ signal) or when *x*_3_ = 1 (i.e., there is a ‘bell’ signal) and *x*_6_+*x*_7_+*x*_8_>1 (i.e., the degree of association is high enough). The degree of association is also updated as the states of *x*_6_, *x*_7_,and *x*_8_ evolve. The value of *x*_6_+*x*_7_+*x*_8_ will remain unchanged (long‐term memory) if *x*_6_ = *x*_7_ = *x*_8_ = 1. Otherwise, it will gradually decrease (short‐term memory).

Following the computing process defined in Figure 4c, the evolution of ‘food’, ‘bell’, ‘salivation’, and association degree is given in Figure 4d. The results confirm all the response cases shown in Figure 4a. With the help of the association degree, the long short‐term memory is realized. For case 3, the association degree first increases and then decreases, indicating the formation of short‐term memory and a forgetting process. For case 4, the association degree gradually increases and remains unchanged after reaching the maximum, i.e., long‐term memory is formed. The experimental system of the MRNN is shown in Figure 4e, where the relationship of several typical parts for the schematic diagram in Figure 4c is indicated. The computing process of the MRNN can be understood with the help of that of the mechanical neuron (Figure 1). Video S3 (Supporting Information) shows the experimental computing process of the MRNN in Figure 4e: all four cases are verified and the results match those shown in Figure 4d.

In addition, more advanced MRNN can be realized by including more mechanical bits, connections, and neurons. For example, the memory evolution process can be designed by adding more mechanical bits in the ARC and adjusting the corresponding weights. Different from the linear forgetting process of the association degree shown in Figure 4d, a biomimetic forgetting process of the memory is designed in the Supplementary Information: the forgetting speed is very fast in the initial stage and then slows down gradually; within a certain duration, the memory of things is kept at a relatively stable level.

## 3 Conclusion

In this paper, an explicit mechanical neuron equivalent to the McCulloch–Pitts model is proposed, serving as the basic computing module of MNNs. A training method is also proposed to enhance the robustness of the neurons. With the explicitness and robustness of the mechanical neuron, two robust MNNs based on more advanced ANN models are designed without the need of solving equilibrium equations. The first is the MCNN, which can still successfully perform handwritten digit recognition even if some constituent springs are removed. The second is the MRNN with long short‐term memory capabilities, mimicking Pavlov's dog that learns in real‐time.

Note that the MNNs in the paper are trained with an offline learning strategy where training is performed on an external computer. Although the weights are fixed during in‐life service, the MNNs trained by offline learning are advantageous in terms of size, power consumption, and cost, among others. In the future, the non‐adjustable springs can be replaced by adjustable ones, as suggested in Ref,^[^ ^40^ ^]^ to further support continued online learning, thus facilitating evolving tasks.

For applications, the MNNs can serve as a mechanical skeleton with neuromorphic intelligence to be embedded in various devices and further expand the functionality of mechanical systems. Robotics with integrated sensing‐analyzing‐actuating intelligence can be made. The sensor can be the buckled beam here for pressure sensing or other stimuli‐responsive materials (e.g., liquid crystal elastomer^[^ ^24^ ^]^) to sense environmental information like heat or light. The actuators can be a magnet or conductive super‐coiled polymer actuators^[^ ^51^ ^]^ for more robust actuation. By choosing other types of explicit mechanical neurons, the concept in this paper can also be applied to other mechanical platforms. For instance, the mechanical neuron shown in Figure S3b (Supporting Information) can be combined with soft robotics for better environmental adaptability, benefited from the development of pneumatic logic devices,^[^ ^11^ ^]^ oscillators,^[^ ^52^ ^]^ and so on. Efforts can be made to extend the developed conception of explicit and robust mechanical neurons to passive systems in the future. In doing so, zero‐power neuromorphic computing applications^[^ ^53^ ^]^ may become possible with the corresponding MNNs. In general, it is expected that this work can serve as a prototype for the key elements of constructing MNNs (i.e., design of explicit mechanical neurons, analysis of weight space, training method informed of the robustness, and construction of advanced network structure) and promote the development of mechanical systems with neuromorphic intelligence.

## 4 Experimental Section

### Mechanism of Buckled Beamed‐Based Mechanical Neuron

The buckled beam was obtained by pre‐compressing a straight beam (Figure S1a, Supporting Information), where *L*, *t*, and *b* are the respective length, thickness, and width of the beam and *d*_0_ is the pre‐compressing distance. The deflection as a function of position *x* is given by *W*(*x*). The beam was made of thermoplastic polyurethanes (TPU). Its elasticity modulus is 81.4 MPa. The two ends of the beam were clamped. During computing, its midpoint was horizontally loaded by a force *F* while the rotation of the midpoint was constrained. The theoretical force‐displacement response of the buckled beam is given by.^[^ ^54^ ^]^ The theory results were compared with the finite element method (FEM) simulation in Figure S1b (Supporting Information), where *W*_0_ is the initial deflection of the midpoint, *W*_0_ − *W*(*L*/2) is the displacement of the midpoint, *L* = 25mm, *t* = 2mm, *b* = 2.5mm, and *d*_0_ = 1.0, 1.2, or 1.5 mm. It can be seen that the numerical and theoretical results are in good agreement. In the study, the buckled beam with *d*_0_ = 1.2 mm is adopted unless otherwise mentioned. Other choices of the geometrical parameter were also possible, as long as the corresponding beam was bistable. More detailed theory analysis of the buckled beam is given in the Supplementary Information. For each of the three force‐displacement response curves, two stable states, i.e., *F* = 0, and the slope of the curve greater than 0, can be identified. The buckled beam can thus be used as a non‐volatile mechanical memory unit^[^ ^6^ ^]^ to store the binary bits 0 or 1 as the beam stays at different stable states, i.e., arching to the left or right. The stored binary bits will be used as the inputs and outputs of the corresponding mechanical neuron.

To realize a nonlinear activation operation, the buckled beam used for the output of the neuron was compressed by pushing a spring with a prescribed displacement *U* (Figure S1c, Supporting Information). The relationship between *U* and W0−W(L/L22) can be obtained theoretically as shown in Figure S1d (Supporting Information) with different spring stiffness *k*. In this paper, the displacement load is set as *U* = 2*W*_0_. The horizontal dash line (*U* = 2*W*_0_) is tangent to the red line with *k* = 0.514 N mm^−1^ in Figure S1d (Supporting Information). If *k* < 0.514 N/mm, e.g., 0.4 N mm^−1^ for the black line in Figure S1d (Supporting Information), the buckled beam remains in state 0 under the load 2*W*_0_. If *k* > 0.514 N mm^−1^, e.g., 0.7 N mm^−1^ for the blue line in Figure S1d (Supporting Information), the buckled beam snaps to state 1. Thus, a critical stiffness for state changing of the buckled beam can be obtained and denoted by k* = 0.514 N mm^−1^. Accordingly, the relationship between the stiffness of the compression spring (*k*) and the state of the output beam (*O*) can be written as *O* = ε(*k* − k*) with the step function (ε(*x*) = 0, *x* < 0;ε(*x*) = 0, *x* ≥ 0), which functions as nonlinear activation for the mechanical neuron.

A series of parallel springs were connected to the output buckled beam to realize the weighted summation operation, Figure 1a. The weights were defined as the dimensionless stiffness of the springs, i.e., *w_i_* = *k_i_*/k*. Then, with the inverters, the weight space can be extended to include both positive and negative values.

To reduce manufacturing costs, a series of standard, commercially available springs were used. The measured geometrical parameter dependence of the spring stiffness is shown in Figure S1e (Supporting Information) with the error bar being the triple standard deviation and *d*, *N*, and *D* the wire diameter, number of spring coils, and outer diameter, respectively. Theoretically, *k* = *Gd* ^4^/[8*N*(*D* − *d*)^3^] where *G* is the shear modulus of the spring.

By adding an additional input *I_b_* = 1 and the corresponding spring *k_b_*, a bias can be introduced to the mechanical neuron. However, the compression strength applied to the output beam is either Ibkb/Ibkbk∗k∗ or (1−Ib)kb/(1−Ib)kbk∗k∗. The bias can only be greater than 0 by setting *I_b_* = 1. To introduce a bias of negative value, an additional spring (*k_g_*) can be added between the output buckled beam and the ground (Figure S1f, Supporting Information). Typical theoretical curves in terms of *U* versus W0−W(L/L22) with different *k* and *k_g_* are given in Figure S1g (Supporting Information). All the curves were tangent to the straight dash line (*U* = 2*W*_0_), indicating that the critical stiffness k* for state changing is a function of *k_g_*, i.e., k* = k*(*k_g_*). For instance, k*(0.173) = 0.576 N/mm and so on. The grounded spring increases k* by Δk* = k*(*k_g_*) − k*(0) and inhibits the state changing of the output beam, which is equivalent to introducing a negative bias to the neuron, b=−Δk∗/−Δk∗k∗(0)k∗(0). Other methods for introducing negative bias were also possible, e.g., the critical stiffness k* can be increased by changing the geometry of the buckled beam, see Figure S1h (Supporting Information). It can be found that k* = 0.644 N/mm when *d*_0_ = 1.3 mm, with Δk* = 0.13 N mm^−1^. Both methods will be adopted in the study to form the bias space with discrete values kb/kbk∗k∗ and −Δk∗/−Δk∗k∗k∗.

Now, the forward process of the buckled beam based neuron can be written explicitly as Equation (1).

### Training Robust MNN by Selecting the Weights and Biases from a Series of Specific Discrete Values

The training process was conducted in Matlab. For the network shown in Figure S5 (Supporting Information), this process is as follows. The forward procedure of the MNN can be written as:

(4) $$a_{j}^{l+1}=\sum_{i=1}^{N_{l}}w_{ij}^{l}I_{i}^{l}-\sum_{w_{ij}^{l}<0}w_{ij}^{l}+b_{j}^{l+1}-1$$

(5) $$I_{j}^{l+1}=\varepsilon \left(a_{j}^{l+1}\right)$$

where *N_l_* is the number of nodes in the *l*‐th layer, $I_{i}^{l}$, $w_{ij}^{l}$, $b_{j}^{l+1}$ and $a_{i}^{l+1}$ are the value of nodes, weight, bias, and weighted summation, respectively, *i*, *j*, and *l* are the input, output and layer index of the corresponding neuron, and $w_{ij}^{l},b_{j}^{l+1}\in D$, with D being a set composed of discrete values.

In the training process, there were two loss functions. The first is defined as:

(6) $$L_{1}=\frac{1}{2}{\left(I_{1}^{n+1}-I_{t}\right)}^{2}$$

where *n* + 1 is the number of layers and *I_t_* is the target output. By minimizing *L*_1_, the network can acquire the target input‐output relationship. The second loss function is defined as:

(7) L2il=ail−r2ail−r22,0<ail≤r2,0<ail≤rail+r2ail+r22,−r<ail≤02,−r<ail≤00,ail≥r

(8) $$L_{2}=\frac{1}{2}\sum_{l=2}^{n+1}\sum_{i=1}^{N_{l}}L_{2i}^{l}$$

where *r* is the robustness parameter. After minimizing *L*_2_, the larger *r*, the more robust the network, as explained in the main text.

Two training phases were being alternately conducted in the training process. In each training phase, the weights and biases were updated along the gradient descent direction of the corresponding loss function to search the target weight in a set of discrete values (i.e., D) until reaching a maximum number of iteration steps set as 200. Note that the gradient of the step function will be replaced by the following function to facilitate training,

(9) $$I_{i}^{l}=\frac{\tanh\left(a_{i}^{l}\right)+1}{2}$$

In the first training phase, the initial weights and biases were first discretized by converting them to the closest discrete value in the set D. The error signal during the backward step can be obtained as:

(10) $$\delta_{i}^{l}=-\frac{\partial L_{1}}{\partial a_{i}^{l}}=\left\{\begin{array}{c} \frac{1}{2}\left(1-\tanh^{2}\left(a_{i}^{n+1}\right)\right)\left(I_{i}^{n+1}-I_{t}\right),l=n+1\\ \frac{1}{2}\left(1-\tanh^{2}\left(a_{i}^{l}\right)\right)\sum_{j=1}^{N_{l+1}}w_{ij}^{l+1}\delta_{j}^{l+1},l\leq n \end{array}\right.$$

Incremental updates of the weight and bias in the first training phase were adopted:

(11) $$\Delta_{1}w_{ij}^{l}=-\eta \frac{\partial L_{1}}{\partial w_{ij}^{l}}=-\eta \frac{\partial L_{1}}{\partial a_{j}^{l+1}}\frac{\partial a_{j}^{l+1}}{\partial w_{ij}^{l}}=\left\{\begin{array}{c} \eta \delta_{j}^{l+1}I_{i}^{l},w_{ij}^{l}\geq 0\\ \eta \delta_{j}^{l+1}\left(I_{i}^{l}-1\right),w_{ij}^{l}<0 \end{array}\right.$$

(12) $$\Delta_{1}b_{j}^{l+1}=-\eta \frac{\partial L_{1}}{\partial b_{j}^{l+1}}=-\eta \frac{\partial L_{1}}{\partial a_{j}^{l+1}}\frac{\partial a_{j}^{l+1}}{\partial b_{j}^{l+1}}=\eta \delta_{j}^{l+1}$$

where η is the learning rate set as 0.1.

As for the second training phase, *L*_2_ is minimized by minimizing all $L_{2i}^{l}$, with the increments in weight and bias given by:

(13) $$\Delta_{2}w_{ij}^{l}=-\eta \frac{\partial L_{2i}^{l+1}}{\partial w_{ij}^{l}}=\left\{\begin{array}{c} \eta \left(a_{j}^{l+1}-r\right)\left(I_{i}^{l}+\varepsilon \left(w_{ij}^{l}\right)-1\right),0<a_{j}^{l+1}\leq r\\ \eta \left(a_{j}^{l+1}+r\right)\left(I_{i}^{l}+\varepsilon \left(w_{ij}^{l}\right)-1\right),-r<a_{j}^{l+1}\leq 0\\ 0,\left|a_{j}^{l+1}\right|\geq r \end{array}\right.$$

(14) $$\Delta_{2}b_{j}^{l+1}=-\eta \frac{\partial L_{2i}^{l+1}}{\partial b_{j}^{l+1}}=\left\{\begin{array}{c} \eta \left(a_{j}^{l+1}-r\right),0<a_{j}^{l+1}\leq r\\ \eta \left(a_{j}^{l+1}+r\right),-r<a_{j}^{l+1}\leq 0\\ 0,\left|a_{j}^{l+1}\right|\geq r \end{array}\right.$$

These two training phases were alternately conducted until the two loss functions were minimized to 0 (e.g., Figure 2c) and the target weights and biases were obtained.

### Fabrication of the Mechanical Model

The buckled beams and computing structures were all designed in the CAD software Solidworks (Dassault Systèmes) and exported as STL files to be used in the subsequent 3D printing. The buckled beam was made of thermoplastic polyurethanes (TPU) and printed using the fused deposition modeling technique on an Ultimaker S3 printer. The springs were made of spring steel. Other components (the links, sliders, supports, and so on) were all made of photosensitive resin (DSM IMAGE8000) and printed using a Stereolithography Apparatus (SLA) UnionTech LT_450_409_G 3D printer. The mechanical computing systems were obtained by assembling all the components. Below the support of each buckled beam, there was a connected electromagnet KK‐1050B (Kakcom) that provides the time signal (periodic external force) under the control of a microcontroller. Besides, petroleum jelly was applied to the surface of all the components to minimize friction.

## Conflict of Interest

The authors declare no conflict of interest.

## Author Contributions

C.Q.C. designed and supervised the research. T.M. proposed the conception, carried out the structural design, coding and experimental work. C.Q.C, T.M, and Y.Z. wrote the manuscript and designed the figures. All authors commented on the paper.

## Supporting information

Supporting Information

Supplemental Video 1

Supplemental Video 2

Supplemental Video 3

## Data Availability

The data that support the findings of this study are available from the corresponding author upon reasonable request.
