# Supplementary material for: Mechanical Neural Networks with Explicit and Robust Neurons
Source: Adv Sci (Weinh). 2024 Jun 19;11(33):2310241. doi: 10.1002/advs.202310241 (PMC11434013; doi:10.1002/advs.202310241)
Supplement: Supplementary file 1 — Supporting Information [file ADVS-11-2310241-s002.pdf]

ADVANCED SCIENCE
Open Access

Supporting Information

for *Adv. Sci.*, DOI 10.1002/advs.202310241

Mechanical Neural Networks with Explicit and Robust Neurons

*Tie Mei, Yuan Zhou and Chang Qing Chen**

Supporting Information

Mechanical neural networks with explicit and robust neurons

*Tie Mei, Yuan Zhou, Chang Qing Chen**

The PDF file includes:

Text

Figs. S1 to S5

Legends for Videos S1 to S3

Other Supplementary Material for this manuscript includes the following :

Videos S1 to S3

Mechanism of the buckled beam with a spring

For the bi-stable buckled beams used to record binary bits 0 and 1, the force-compression relationship is given in Ref 51 as:

$$f(\eta)=4\sqrt{\frac{E^2I^2\eta^5\left(d_0-d_p(\eta)\right)}{L^5\left(2\eta-12\tan\left(\frac{\eta}{4}\right)+\eta\sec^2\left(\frac{\eta}{4}\right)\right)}} \tag{1}$$

$$W_m(\eta)=-\frac{L^3}{EI\eta^2}\left(\frac{1}{4}-\frac{1}{\eta}\tan\left(\frac{\eta}{4}\right)\right)f(\eta) \tag{2}$$

where η is a parameter larger than 2π, $W_m(\eta)$ is the midpoint deflection of the beam, $f(\eta)$ is the reaction force, E is the elasticity modulus, $I=bt^3/12$, and $d_p(\eta)=\eta^2I/(Lbt)$. Note that when η reaches 4π the deflection response lies between that predicted by the parametric equation (1) and a lower reaction force given below,

$$f(W_m)=-\frac{64\pi^2EIW_m}{L^3} \tag{3}$$

Following equations (1) to (3), the theoretical results in Figure S1b are obtained. The numerical results are obtained by the finite element method (FEM) using the commercially available software ABAQUS, with the static general module. The buckled beam is meshed with 21 Timoshenko beam elements (B21), and the Poisson's ratio of the beam is set as 0.45 by considering TPU is nearly incompressible.

In addition, the relationship between displacement load U and the deflection of the beam's midpoint $W_0-W(L/2)$ in Figure S1d can be written as:

$$U=\frac{f\left(W(L/2)\right)+k_g\left(W_0-W(L/2)\right)}{k}+W_0-W(L/2) \tag{4}$$

With this equation, the theory results in Figure S1g, h can be obtained for different k and k_g.

Assembling of the mechanical neuron based on buckled beam

The assembling details of the mechanical neuron in Figure 1 are shown in Figure S2. For the components related to input 1, slider blocks 1 and 2 are fixed in the slider groove 1 and can only move translationally along the *x*-direction. Slider blocks 2 and 3 are fixed in the slider grooves 2 and 3, respectively. They can only move translationally along the *y*-direction. For the components related to input 2, slider blocks 5 and 6 are fixed in slider groove 4 and can only move translationally along the *x*-direction. Slider blocks 7 and 8 are fixed in slider grooves 5 and 6, respectively, where only translational motion along the *y*-direction is allowed. As for the output-related components, slider blocks 9, 10 11, 12, and 14 are fixed in slider grooves 1, 4, 7, 8, and 9, respectively. Slider block 13 is fixed in slider grooves 7, 8, and 9. All these blocks can only move translationally along the *x*-direction. In addition, the midpoints of the buckled beam in input 1, input 2, and the output are fixed in slider blocks 1, 5, and 14. Thus, their rotation is prohibited. The ends of all the buckled beams are inserted into the corresponding slots and are clamped.

Other designs of the mechanical neuron

In Figure S3a, a slider on a rough horizontal plane is pushed by a series of parallel springs, where k_i is the stiffness of the *i*-th spring. The input I_i is the displacement load applied to the springs while the output O is the displacement of the slider. The slider slides only when the force applied to the slider is larger than the maximum static friction f_s. After sliding, the equilibrium equation can be written as $\sum_{i=1}^{n} k_i \left(I_i - O\right) = f_\mathrm{s}$. By considering the weights as $w_i = k_i \big/ \sum_{j=1}^{n} k_j$ and the bias as $b = f_\mathrm{s} \big/ \sum_{i=1}^{n} k_i$, the output can be written as a ReLU function ($\mathrm{Relu}\left(x\right) = \max\left(0, x\right)$) of the weighted summation of the inputs (Figure S3a, below). Here, the weighted summation operation is derived from the parallel arrangement of springs and the ReLU operation is realized with the help of the static friction f_s.

In addition, a mechanical neuron composed of the intersecting water pipes is shown in Figure S3b. The inputs are the displacement of the pistons in the left water pipes and d_i is the inner diameter of the *i*-th pipe. The pipes are filled with water (shown as the blue area) except for the gap of length $\overline{I}$ in the right pipe of inside diameter d_o. When the left pistons move, the slider also moves, driven by water. There is no pressure in the chamber of the gap because of the venthole. Only after moving across the gap and touching the right piston, will the slider

push the piston for a distance of O as the output. The change in the volume of water for the left and right pipes is the same considering the water is incompressible, i.e., $\sum_{i=1}^{n} 0.25\pi d_i^2 I_i = 0.25\pi d_{\text{o}}^2 \left(\overline{I} + O\right)$. Setting the weight and bias as $w_i = d_i^2 / d_{\text{o}}^2$ and $b = \overline{I}$, respectively, these intersecting water pipes can serve as a mechanical neuron as the input-output relationship listed in the below Figure S3b.

Design the memory evolution of the mechanical Pavlov's dog

An advanced output gate with more memory units in the ARC is constructed to mimic a more complex process of memory evolution (schematically shown in Figure S4). In each work period, x_1 receives the association judgment result from the input gate, e.g., $x_1 = y_{t^*}$ at the t^*-th period where y_{t^*} is the corresponding association judgment result, and x_{N+1} also receives the 'bell' signal. Then, x_1 to x_N evolve with the help of the shifter register operation, i.e., $x_{i+1} = x_i$, i=1, 2, …, N, and the output is also obtained as: $x_{N+2} = \varepsilon\left(\sum_{i=1}^{N} w_i x_i + w_{N+1} x_{N+1} - 1\right)$. Under the evolution rule, $x_{t-t^*+1} = y_{t^*}$ at the t-th period, i.e., the association judgment result of the t^*-th period is stored in the x_{t-t^*+1} at the t-th period. Thus, we define the memory degree of y_{t^*} at the t-th period as:

$$m\left(y_{t^*}, t\right) = w_{t-t^*+1} x_{t-t^*+1} = w_{t-t^*+1} y_{t^*} \qquad (5)$$

Accordingly, it is possible to design the evolution of the memory by setting the weights. For example, to realize a biomimetic forgetting process, i.e., the forgetting speed is very fast in the initial stage, and then slows down gradually; at a certain time, the memory of things is kept at a relatively stable level and the weights can be set as $w_i = e^{-c(i-1)} w_1$. Thus, the memory evolution can be written as:

$$m\left(y_{t^*}, t\right) = e^{-c\left(t-t^*-1\right)} w_1 y_{t^*} \qquad (6)$$

where c denotes the rate of memory decay.

List of supplementary figures

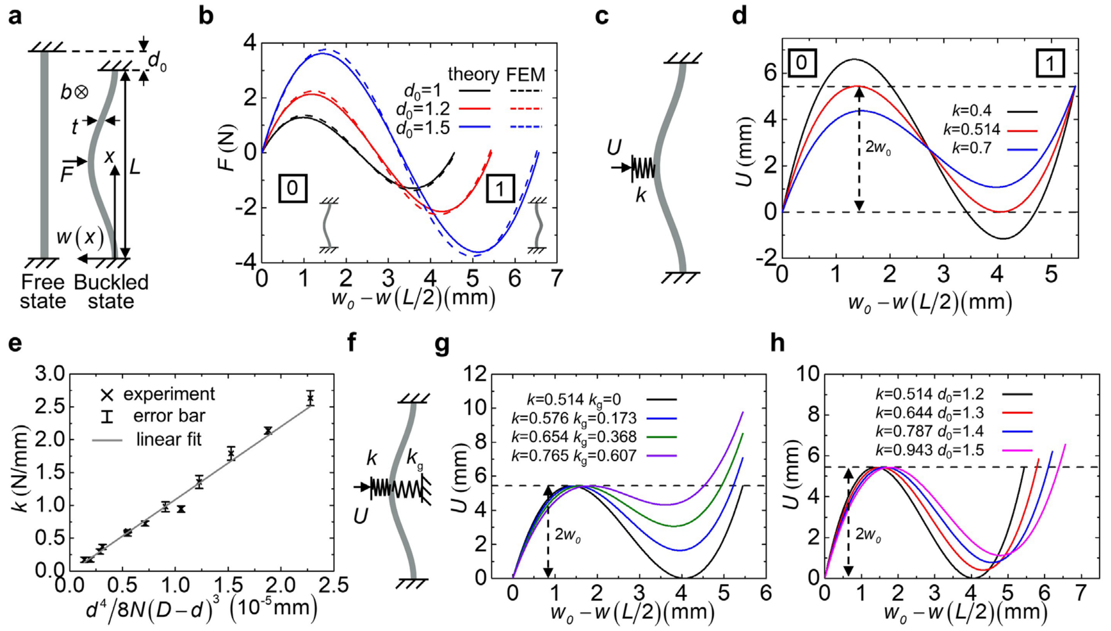

Figure S1 Mechanism of buckled beamed based mechanical neuron. (**a**) Geometry of the buckled beam. (**b**) FEM and theoretical results of the force-displacement curves of the buckled beam in (**a**). (**c**) A buckled beam loaded by displacement U through a connecting spring. (**d**) The relationship between the compressing displacement U and the deflection of the beam's midpoint. (**e**) Measured stiffness of the springs. (**f**) A buckled beam with a grounded spring is compressed via a connecting spring. (**g**) and (**h**) The relationship between the compressing displacement and the deflection of the beam's midpoint with different stiffnesses of the grounded springs (and pre-compressing distance).

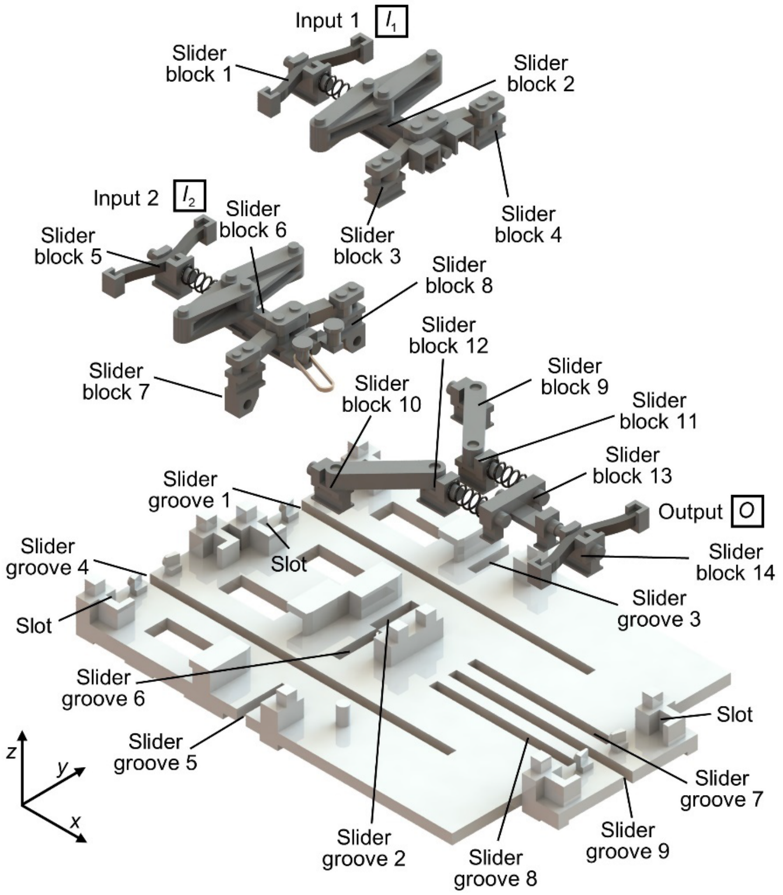

Figure S2 Assembling of the mechanical neuron based on buckled beam.

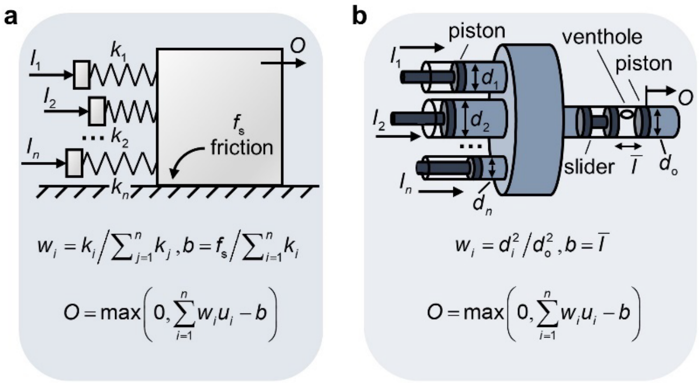

Figure S3 Other designs of mechanical neurons. (**a**) Mechanical neuron composed of sliders under coulomb friction and parallel springs. (**b**) Mechanical neuron composed of intersecting water pipes.

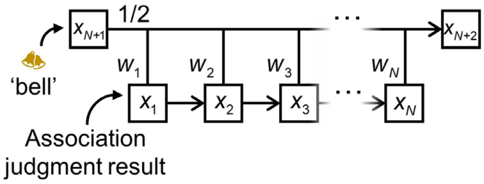

Figure S4 Design of advanced memory evolution process.

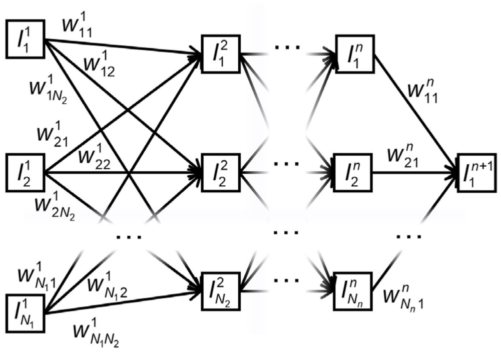

Figure S5 A schematic mechanical neural network.

Supplementary Videos

Video S1. A mechanical convolutional neural network for handwritten digits recognition.

(1) The trained mechanical convolutional neural network can recognize handwritten digits 0s and ls.

(2) The trained mechanical convolutional neural network can recognize unseen handwritten digits 0s and 1s.

Video S2. Even if some springs are removed, the trained mechanical neural network can still successfully recognize handwritten digits 0s and ls.

Video S3. A mechanical recurrent neural network with long short term memory for associative learning.

(1) The "dog" salivates when it sees food.

(2) The "dog" does not salivate when the bell rings.

(3) The "dog" gradually learns the relationship between the food and the bell. Then, it salivates when the bell rings. However, the learned knowledge only converts to short-term memory because of the short learning time. The "dog finally does not salivate with the bell ringing as time passes.

(4) The "dog" gradually learns the relationship between the food and the bell. The learned knowledge converts to long-term memory for a long learning time. The "dog" salivates with the bell ringing no matter how long time has passed.
